# Meditation-Specific Neural Predictors of State Mindfulness During Eyes Open Meditation

**DOI:** 10.1007/s12671-026-02850-6

**Published:** 2026-05-11

**Authors:** Marne L. White, Yanli Lin, Todd S. Braver

**Affiliations:** 1 University of Maryland, College Park, College Park, United States; 2 University of Arkansas at Fayetteville, Fayetteville, United States; 3 Washington University in St. Louis, St. Louis, United States

**Keywords:** EEG, Mindfulness, Meditation, Alpha, Theta

## Abstract

**Objectives:**

Focused attention (FA) and open monitoring (OM) are distinct mindfulness practices that produce unique psychological effects. Prior research has frequently investigated the neural correlates of FA and OM states utilizing electroencephalographic (EEG) methods, focusing on changes in spectral power within theta and alpha bands. Yet the functional significance of these neural changes has remained unclear. Here we utilized a fully within-subject state induction protocol to more directly test whether EEG spectral power during FA and OM is differentially associated with subjective ratings of state mindfulness.

**Method:**

While continuous EEG was recorded, participants engaged in eyes-open audio-guided FA and OM practices, as well as an active control condition (C), and then self-reported their state mindfulness afterwards. Linear mixed-effects models were used to rigorously assess how condition-level variation in spectral power predicted state mindfulness scores across the three inductions.

**Results:**

Validating the approach, participants reported higher state mindfulness and decreased theta power during both FA and OM relative to C; additionally, reduced alpha power was found in OM relative to FA. Most importantly, increased theta power was associated with higher state mindfulness in FA and even more strongly in OM, whereas reduced alpha power was linked to higher state mindfulness selectively in OM.

**Conclusions:**

These findings add to the growing literature suggesting that FA and OM represent distinct mindfulness states. We further establish that the functional significance of theta and alpha power is context-dependent, clearly linking these neural measures to the subjective quality of specific meditation states and practices.

**Supplementary Information:**

The online version contains supplementary material available at 10.1007/s12671-026-02850-6.

A foundational goal within the maturing field of contemplative neuroscience is to bridge the gap between subjective, first-person experience, and objective, physiological measurement (Varela et al., 2017; Wallace, 2007). One central focus of investigation involves identifying reliable neural markers that correspond to the cultivation of mindfulness, defined as a state of nonjudgmental awareness and acceptance (Bishop et al., 2004; Kabat-Zinn, 1990). Toward this end, electroencephalography (EEG) has proven valuable (see Lin et al., 2025 for an overview), with much previous research focusing on oscillatory activity occurring within the theta (4–8 Hz) and alpha (8–13 Hz) frequency bands (Cahn & Polich, 2006; Lomas et al., 2015). Within the broader cognitive neuroscience literature, theta power has been linked to cognitive control processes (Cavanagh & Frank, 2014; Eisma et al., 2021), whereas enhanced alpha activity (i.e., increased power) has been associated with internally directed attention and suppression of irrelevant task stimuli (Cooper et al., 2003; Foxe & Snyder, 2011). Together, this has led to the compelling and pervasive suggestion that specific patterns of alpha and theta activity may represent a unique neural signature of meditation, such as the state of “relaxed alertness” described by Lomas et al. (2015).

With that said, progress toward validating this intuitive idea has been impeded by a lack of conceptual cohesion and methodological standardization, which has produced mixed findings. For example, although alpha activity has been reliably linked to mindfulness, some studies report increased alpha power while others find no effect or even decreases, with large variation in the type of mindfulness practice and expertise level of the sample (Cahn & Polich, 2006; Lee et al., 2018; Lieberman et al., 2025; Lomas et al., 2015; Rodriguez-Larios et al., 2021). Indeed, these discrepancies reflect the core challenge that mindfulness is not a unitary construct, but rather encompasses a family of distinct meditation practices and corresponding psychological states (Davidson & Kaszniak, 2015; Lin et al., 2022; Van Dam et al., 2018). For example, the neurocognitive demands of focused attention (FA), which involves sustaining attention in the midst of distraction, are fundamentally different from those of open monitoring (OM), which involves cultivating a broader state of nonjudgmental awareness of arising experience (Lutz et al., 2008).

This distinction between these practices underscores the key point that the phenomenological characteristics and functional significance of theta and alpha power are likely to be *state dependent*. Such ambiguity leaves the interpretation of neural signals vulnerable to the classic problem of reverse inference, where brain activity patterns are used to infer a “mindfulness” state without explicitly accounting for, or directly manipulating, the specific practice and associated experiential state that is engaged. Thus, although a growing body of work has supported that FA and OM are neurally and functionally dissociable (Brown et al., 2022; Garcia et al., 2024; Lin et al., 2024a, b; Lohani et al., 2020; Manna et al., 2010; Marchand, 2014), few studies have directly tested how brain activity differs while engaged in different mindfulness practices (Garcia et al., 2024; Katyal & Goldin, 2021; Lin et al., 2026; Reggente et al., 2025). This progress has solidified the critical need to ground functional interpretations of mindfulness-related neural oscillations to the subjective features of these distinct meditation states.

To address this need, our group recently completed a study specifically developed to parse the influence of discrete mindfulness states in a sample of novice meditator participants. Importantly, prior analyses from this project supported the validity of the state induction protocol, yielding dissociable transfer effects of FA and OM onto cognitive control and emotional reactivity (i.e., during immediate subsequent task performance; Lin et al., 2024a, b). The study utilized a fully within-subject, multi-session design in which participants completed three EEG-recorded testing sessions under FA, OM, or an active control (C) condition. In each session, participants completed a 10-min guided audio induction to cultivate the target state (guided meditations for the FA and OM conditions, and an educational TED talk for C), prior to performing the Eriksen flanker and affective picture viewing task. Crucially, continuous EEG activity was recorded during each state induction, while self-report ratings on the subjective quality of the practice were collected immediately following its completion, using the psychometrically validated State Mindfulness Scale (SMS; Tanay & Bernstein, 2013). Thus, this design provided a powerful means to directly test the association between real-time neural oscillatory activity and the subjective quality of FA and OM meditative states.

The aim of this paper is to provide a “proof-of-concept” approach for grounding the functional significance of spectral power during theoretically distinguishable meditation practices and states. By modeling alpha and theta power as independent predictors of state mindfulness scores, we can determine if these neural signatures are differentially associated with subjective appraisals of FA and OM. We contrasted this subjectively grounded approach with the more traditional one, in which the analytic comparisons involve only neural measures, to provide a concrete illustration of how state-dependent variability in spectral power can be differentially related to the subjective quality of each distinct mindfulness practice. Demonstrating that these foundational mind-brain relationships can be reliably dissociated in novices provides a critical anchor point to guide the future of our work and possibly the field more broadly.

## Method

### Participants

Thirty native or fluent English-speaking participants with no previous mindfulness experience were recruited using flyers, email, and word-of-mouth advertising. One participant was excluded from all data analyses due to repeated failure to comply with task instructions (e.g., falling asleep during the inductions and clicking randomly during tasks). The final sample consisted of 29 participants (18–35 years old, *M* = 20.72, *SD* = 4.04; 17 females, 12 males) totaling 87 sessions of data which exceeded the preregistered number of 28 participants needed to detect the hypothesized within-subject interaction effects for the parent study with 0.80 power (assuming medium effect size of *d* = 0.50). Two participants did not complete the manipulation check measures during one session, one due to experimenter error (forgetting to administer the survey) and another due to technical failure (data collection server was down). Participants were compensated US$80 for full completion of the study.

### Procedure

The study protocol consisted of three random-order EEG testing sessions, each lasting approximately 2 h and occurring on separate days, involving open monitoring (OM), focused attention (FA), or active control (C) state inductions. At the beginning of the first testing session, participants completed a self-report battery containing demographic information and trait measures. After completion of EEG setup, each session began with a 10-min guided audio induction of OM, FA, or C, followed immediately by performance of the picture viewing or flanker task (task order randomized). Participants then listened to the same audio induction prior to completing the second task (i.e., participants completed the induction audio twice per session). Sessions ended with participants completing a series of manipulation check survey questions that assessed state mindfulness during the audio inductions and probed for the subjective quality of the guided practice as well as their engagement and responsivity to the inductions.

### Measures

#### Audio Inductions

All audio inductions were exactly 10 min in length. The FA and OM inductions were recorded by a certified Mindfulness-Based Stress Reduction instructor and used by our group in other work (Lin et al., 2024a, 2024b, 2026; Tang & Braver, 2020; White et al., 2024). Briefly, the FA induction instructed participants to sustain attention to the breath and to redirect attention back to the breath anytime they noticed mind wandering. The OM induction-guided participants to bring attention to any arising thoughts, feelings, and bodily sensations in an open, nonjudgmental manner. The C induction was a duration-matched audio recording of a TED talk by the linguist Chris Lonsdale (TEDx Talks, 2013) explaining how to rapidly acquire second language proficiency, which was also used in prior studies (Lin et al., 2016, 2019, 2024a, b, 2026). Participants were instructed to keep their eyes open during the audio inductions to mitigate sleepiness, which has been shown to be selectively increased by mindfulness inductions (Lin et al., 2020).

#### State Mindfulness

State mindfulness was measured with the 21-item State Mindfulness Scale (SMS; Tanay & Bernstein, 2013), which uses a 5-point Likert scale ranging from 1 (*not at all*) to 5 (*very well*), to assess momentary awareness of thoughts, emotions, and physical sensations. Participants completed this questionnaire at the end of each session and were directed to fill out the items in relation to the quality of their practice during the guided FA and OM meditations and C audio.

#### Manipulation Check

A manipulation check questionnaire, previously used in our lab (Lin et al., 2024a, b) that assessed engagement and reception to the audio inductions, was administered at the end of each session. Participants were asked to rate the extent to which they found the audio engaging, interesting, and arousing (*1* = *not at all*,* 7* = *very*). Participants also indicated their emotional reaction (*1* = *very negative*, *4* = *neutral*, *7* = *very positive*), comprehension level (*1* = *did not understand*, *7* = *completely understand*), and whether they learned anything (*1* = *very little, 7* = *very much*), as well as their physical comfort (*1* = *not comfortable*, *7* = *very comfortable*). Lastly, participants reported their sleepiness level (*1* = *feeling active, vital, alert, or wide awake*, *8* = *I fell asleep*), during the audios using the Stanford Sleepiness Scale (Hoddes et al., 1973)*.*

### Data Analyses

#### EEG Recording and Preprocessing

Participants were fitted with a 64-channel Lycra EEG cap, and continuous EEG activity was recorded using a Brain Vision actiCHamp Plus system (Brain Vision LLC, Morrisville, NC). Recordings were taken from 32 Ag-AgCl electrodes placed in accordance with the 10/20 system with a sampling rate of 500 Hz. Horizontal and vertical electrooculogram activity was recorded using three electrodes placed around the eyes, two lateral to the outer canthi of each eye and one directly under the right pupil and below electrode site Fp2.

Offline analyses were conducted using BrainVision Analyzer 2 (BrainProducts, Gilching, Germany). Data recorded during the guided audio inductions were band-pass filtered between 0.3 and 50 Hz and then re-referenced to the average of all scalp electrodes (i.e., common average reference). Ocular artifacts were detected and corrected using the regression approach developed by Gratton et al. (1983). Data was portioned into 2-s epochs and then subjected to automatic artifact rejection. Specifically, a computer algorithm detected and removed trials with at least one electrode that met the following criteria: a maximum voltage step of more than 50 μV between sample points, a voltage difference of more than 300 μV within 200 ms intervals, voltage exceeding ± 200 μV, or a maximum voltage difference less than 0.5 μV within 100 ms intervals.

Consistent with previous work (Lin et al., 2020), a fast Fourier transform was applied to all artifact-free epochs, weighted with a Hamming window that tapers the distal 10% of each epoch. The data were then averaged across epochs, and integrated spectral power was computed for the alpha (8–13 Hz) and theta (4–8 Hz) frequency bands for analyses. Using the regional division outlined in Lagopoulos et al., 2009 and Lin et al., 2020, spectral power at each electrode site was averaged into one of three regions of interest—frontal (Fp1, Fpz, Fz, F3, F7, FC1, FC5, FC2, FC6, F4, F8, Fp2), temporal-central (C3, CP1, CP5, CP2, CP6, C4), and posterior (Oz, P10, P4, P3, P9, PO3, PO4, PO7, PO8, Pz). All values were log-transformed to normalize their distribution.

#### Statistical Analyses and Predictions

The lme4 (Bates et al., 2015) and lmerTest (Kuznetsova et al., 2017) packages in R statistical software were used to conduct frequentist linear mixed-effects regression. A critical aim of these analyses was to examine how alpha and theta spectral power interact with induction condition and scalp region to predict state mindfulness scores. Consequently, we fit a single model that simultaneously examined alpha and theta as predictors, allowing us to test whether neural oscillatory activity within these bands produces independent effects on state mindfulness quality.

As a reference benchmark, and to explicitly contrast our primary analysis with traditional approaches, we first conducted conventional, condition-related analyses. These models treated alpha and theta power as dependent variables to test for direct effects of the audio inductions. The models included induction condition and scalp region as predictors, along with a random intercept for participant, specified in Wilkinson notation as: $Alpha/Theta\;Spectral\;Power\;\sim\;1\;+\;Induction\;Condition\;\ast\;Scalp\;Region\;+\;(1\;\vert\;Subject)$. Critically, this comparative strategy allowed us to establish the overall neural effects of the state inductions in a manner consistent with historical approaches, thereby contextualizing and highlighting the novelty of our primary aim to ground mindfulness related neural signatures in their subjective context.

In contrast with the conventional analysis, our primary regression model of interest treated state mindfulness scores as the dependent variable of interest, and included three-way interactions involving induction condition (C, FA, OM), scalp region (frontal, temporal, posterior), and spectral power (alpha and theta separately). The model is specified as: $SMS\;\sim\;1+\;Induction\;Condition\;\ast\;Scalp\;Region\;\ast\;Alpha\;+\;Induction\;Condition\;\ast\;Scalp\;Region\;\ast\;Theta\;+\;(1\;\vert\;Subject)$ This specification enabled us to test: (1) the extent to which alpha and theta exhibit differentiable relationships with state mindfulness across the different induction conditions (FA vs. OM vs. C); and (2) whether these associations vary across scalp regions, reflecting the possibility that topographic variation may moderate the relationship between spectral power and state mindfulness scores.

Both alpha and theta power were within-subject centered by subtracting each participant’s mean across the three sessions from their respective session-specific power values. This procedure isolates within-subject variance, allowing us to examine how session-related variation in spectral power relates to session-related variation in state mindfulness scores. Induction condition was dummy coded with the active control (C) condition as the referent category to examine how focused attention (FA) and open monitoring (OM) differ from the control induction. Scalp region was effect coded to aid interpretation. Finally, a random intercept for participants was included to account for individual differences in baseline state mindfulness. Taken together, this approach allowed us to test the prediction that alpha and theta power exhibit distinguishable relationships to state mindfulness across FA and OM, and that these patterns may differ across scalp regions. The conventional analyses described above departed slightly from our preregistered analyses in that they did not include trait mindfulness and session order as predictors. This was done both to simplify the models, but more importantly to enable a fairer comparison with the conventional benchmark analyses, by having a consistent set of predictors across both. All data, materials, protocols, and analysis code, including full model specifications and output, are available in the OSF repository.

## Results

Demographic characteristics and the descriptive statistics of the audio manipulation check measures, including the State Mindfulness Scale (SMS), are provided in Tables 1 and 2. To streamline reporting, we describe only main and interactive effects pertinent to the core research question (e.g., induction effects and their interactions with alpha and theta power); however, full model summaries containing all parameter estimates are presented in Tables 3, 4, and 5.

**Table 1:** Demographic information

Variable	*N*
Gender	
Male Female	12 17
Ethnicity	
Not Hispanic or Latino Hispanic or Latino	27 2
Race	
White Black or African American Asian Native Hawaiian or Other Pacific Islander American Indian or Alaskan Native More than one race	13 0 15 1 0 0

**Table 2:** Descriptive statistics of manipulation check responses

	C *N* = 29			FA *N* = 29			OM *N* = 27		
Variable	*Range*	*M*	*SD*	*Range*	*M*	*SD*	*Range*	*M*	*SD*
Audio Engagement*	2–7	4.48	1.60	1–6	3.41	1.38	1–6	3.39	1.34
Audio Interest*	2–7	4.90	1.52	1–5	3.10	1.04	1–6	3.14	1.38
Audio Emotional Reaction*	3–7	4.93	1.13	1–6	4.17	1.17	3–7	4.39	0.99
Audio Arousal*	1–6	3.38	1.78	1–5	2.45	1.21	1–5	2.56	1.25
Audio Understanding*	3–7	5.97	1.09	5–7	6.48	0.69	2–7	5.63	1.28
Audio Learning*	2–7	4.93	1.49	1–6	3.66	1.29	1–7	4.41	1.45
Audio Physical Comfort*	3–7	5.17	1.20	2–6	4.38	1.15	3–6	4.56	1.12
Audio Sleepiness	1–6	3.52	1.60	1–6	4.14	1.53	1–7	4.11	1.62
State Mindfulness Scale *	27–86	54.62	15.02	54–101	71.69	10.87	52–103	74.41	12.04

* denotes significant difference by condition (*F*s > 4.27, *p*s < 0.02)

**Table 3:** Model output for theta power predicted by induction

Model	Fixed effects	Estimate (*SD*)	*t*-value	*p*-value
Theta—C as referent	Intercept	−1.29 (0.09)	−14.84	< 0.001*
	Induction OM	−0.08 (0.02)	−3.68	< 0.001*
	Induction FA	−0.06 (0.02)	−2.52	0.01*
	Region Frontal	0.25 (0.02)	11.30	< 0.001*
	Region Temporal	−0.42 (0.02)	−19.00	< 0.001*
	Region Posterior	0.17 (0.02)	7.70	< 0.001*
	Induction OM:Region Frontal	−0.02 (0.03)	−0.71	0.48
	Induction FA:Region Frontal	−0.03 (0.03)	−0.89	0.37
	Induction OM:Region Temporal	0.03 (0.03)	0.84	0.40
	Induction FA:Region Temporal	0.02 (0.03)	0.63	0.53
	Induction OM:Region Posterior	0.00 (0.03)	−0.13	0.90
	Induction FA:Region Posterior	0.00 (0.03)	0.25	0.80

*FA* focused attention, *OM* open monitoring. * denotes *p*-values < 0.05

**Table 4:** Model output for alpha power predicted by induction

Model	Fixed effects	Estimate (*SD*)	***t***-value	*p*-value
Alpha—C as referent	Intercept	−1.10 (0.15)	−7.35	< 0.001*
	Induction OM	−0.04 (0.03)	−1.51	0.13
	Induction FA	0.04 (0.03)	1.38	0.17
	Region Frontal	−0.01 (0.03)	−0.46	0.65
	Region Temporal	−0.44 (0.03)	−15.02	< 0.001*
	Region Posterior	0.45 (0.03)	15.48	< 0.001*
	Induction OM:Region Frontal	−0.04 (0.04)	−0.94	0.35
	Induction FA:Region Frontal	−0.05 (0.04)	−1.27	0.21
	Induction OM:Region Temporal	0.06 (0.04)	1.41	0.16
	Induction FA:Region Temporal	0.04 (0.04)	1.04	0.30
	Induction OM:Region Posterior	−0.02 (0.04)	−0.47	0.64
	Induction FA:Region Posterior	0.01 (0.04)	0.23	0.83

*FA* focused attention, *OM* open monitoring. * denotes *p*-values < 0.05

**Table 5:** Model output for SMS moderated by alpha and theta power

Model	Fixed effects	Estimate (*SD*)	*t*-value	*p*-value
C as referent	Intercept	5.74 (0.22)	26.66	< 0.001*
	Induction OM	1.66 (0.22)	7.55	< 0.001*
	Induction FA	1.41 (0.20)	7.05	< 0.001*
	Region Frontal	0.46 (0.19)	2.38	0.02*
	Region Temporal	−0.89 (0.22)	−4.02	< 0.001*
	Region Posterior	0.43 (0.20)	2.02	0.04*
	Alpha Power	0.73 (0.35)	2.13	0.03*
	Theta Power	−2.63 (0.43)	−6.15	< 0.001*
	Induction OM:Theta	2.72 (0.74)	3.67	< 0.001*
	Induction FA:Theta	1.46 (0.68)	2.14	0.03*
	Induction OM:Alpha	−1.74 (0.51)	−3.41	< 0.001*
	Induction FA:Alpha	−0.01 (0.49)	−0.02	0.98
	Induction OM:Region Frontal	−0.71 (0.29)	−2.43	0.02*
	Induction FA:Region Frontal	−0.23 (0.28)	−0.82	0.41
	Induction OM:Region Temporal	0.80 (0.36)	2.22	0.03*
	Induction FA:Region Temporal	0.78 (0.34)	2.30	0.02*
	Induction OM:Region Posterior	−0.09 (0.31)	−0.28	0.78
	Induction FA:Region Posterior	−0.56 (0.29)	−1.91	0.06
	Region Frontal:Alpha	0.53 (0.48)	1.10	0.27
	Region Temporal:Alpha	0.89 (0.49)	1.81	0.07
	Region Posterior:Alpha	−1.42 (0.45)	−3.17	< 0.01*
	Region Frontal:Theta	0.15 (0.50)	0.31	0.76
	Region Temporal:Theta	−0.92 (0.57)	−1.61	0.11
	Region Posterior:Theta	0.77 (0.52)	1.46	0.14
	Induction OM:Region Frontal:Theta	0.18 (0.86)	0.21	0.83
	Induction FA:Region Frontal:Theta	0.00 (0.76)	0.00	1.00
	Induction OM:Region Temporal:Theta	1.41 (0.93)	1.52	0.13
	Induction FA:Region Temporal:Theta	0.84 (0.84)	1.00	0.32
	Induction OM:Region Posterior:Theta	−1.59 (0.92)	−1.73	0.08
	Induction FA:Region Posterior:Theta	−0.84 (0.76)	−1.11	0.27
	Induction OM:Region Frontal:Alpha	−1.14 (0.64)	−1.78	0.08
	Induction FA:Region Frontal:Alpha	−0.87 (0.67)	−1.29	0.20
	Induction OM:Region Temporal:Alpha	−0.70 (0.65)	−1.07	0.28
	Induction FA:Region Temporal:Alpha	−0.52 (0.66)	−0.79	0.43
	Induction OM:Region Posterior:Alpha	1.84 (0.62)	2.98	< 0.01*
	Induction FA:Region Posterior:Alpha	1.39 (0.59)	2.34	0.02*

*SMS* State Mindfulness Scale, *FA* focused attention, *OM* open monitoring. Alpha and theta values are within-subject centered, calculated by subtracting the alpha and theta values for each induction and region from the respective, subject-level mean. * denotes *p*-values < 0.05

### Manipulation Check

One-way repeated measures ANOVAs were conducted on each manipulation check measure to assess for condition differences. As shown in Table 2, these analyses revealed significant differences across conditions for all measures except sleepiness. Briefly, post hoc *t*-tests revealed that participants reacted more positively to the C induction than the focused attention (FA) induction (*t*_(28)_ = 2.63, *p* = 0.04), reported higher levels of arousal during the C induction relative to both the open monitoring (OM) and FA inductions (*t*_*(26)*_ = 2.66, *p* = 0.04; *t*_*(28)*_ = 2.49, *p* = 0.04), and reported greater learning in C relative to both OM and FA (*t*_*(28)*_ = 4.18, *p* < 0.001; *t*_*(26)*_ = 2.16, *p* = 0.04). Participants also reported higher interest and physical comfort levels for C than FA (*t*_*(28)*_ = 4.90, *p* < 0.001; *t*_*(28)*_ = 3.24, *p* = 0.01) or OM (*t*_*(26)*_ = 4.46, *p* < 0.001; *t*_*(26)*_ = 2.70, *p* = 0.02).

Consistent with the intent of the experimental manipulation, participants reported significantly higher SMS scores after both the FA (*t*_*(28)*_ = 4.92, *p* < 0.001) and OM (*t*_*(26)*_ = 4.88*, p* < 0.001) inductions compared to the C condition. SMS scores for FA and OM were not significantly different from each other (*t*_*(26)*_ = 1.92, *p* = 0.07). However, participants reported greater levels of understanding during FA relative to both OM and C (*t*_*(26)*_ = 3.35, *p* = 0.01; *t*_*(26)*_ = 2.82, *p* = 0.02), but greater learning during OM relative to FA (*t*_*(26)*_ = 2.55, *p* = 0.03). This intriguing double dissociation already points to subtle differences in subjective experience associated with the two mindfulness practices.

### State Induction Effects on Spectral Power

Conventional benchmark analyses tested whether the audio inductions produced overall changes to spectral power. Both FA and OM were associated with decreased theta power compared to C (FA: *b* = −0.06, *SD* = 0.02,* t* = −2.52, *p* = 0.01; OM: *b* = −0.08, *SD* = 0.02,* t* = −3.68, *p* < 0.001; Table 3), though were not different from each other (*b* = −0.03, *SD* = 0.02,* t* = −1.17, *p* = 0.24). Regarding alpha power, there were no significant main effects or interactions when compared to the C condition (*bs* > 0.04, *p* > 0.13; Table 4); however, a direct comparison between the mindfulness inductions revealed that OM was characterized by lower alpha power than FA (*b* = −0.08, *SD* = 0.03,* t* = −2.89, *p* < 0.01). Although these standard analyses support the hypothesis that FA and OM are neurally differentiable, they do not permit interpretations regarding the functional significance of these differences in spectral activity. Namely, they are fundamentally uninformative about how variability in alpha and theta relates to the subjective quality of mindfulness during these different practices.

### Spectral Power Influences on State Mindfulness

To address this core question, our primary analysis modeled spectral power as a within-subject predictor of SMS scores (computed by subtracting alpha and theta power values for each induction from their respective subject-level mean, Table 5). Mirroring the manipulation check responses, the model revealed significant main effects of induction, with both FA (*b* = 1.41, *SD* = 0.20,* t* = 7.05, *p* < 0.001) and OM (*b* = 1.66, *SD* = 0.22,* t* = 7.55, *p* < 0.001) producing higher SMS scores than C. Importantly though, these effects were qualified by higher-order interactions involving spectral power. For theta power, significant two-way interactions with induction condition were observed, such that greater theta power was associated with higher SMS during FA (*b* = 1.46, *SD* = 0.68,* t* = 2.14, *p* = 0.03) and OM (*b* = 2.72, *SD* = 0.74,* t* = 3.67, *p* < 0.001) relative to C. This was further qualified by a trending three-way interaction involving scalp region, such that the strength of association between theta power and SMS in the posterior region was selectively attenuated during OM relative to C (i.e., although greater theta power was still associated with higher SMS during OM relative to C, this difference was reduced in the posterior region; *b* = −1.59, *SD* = 0.92,* t* = −1.73, *p* = 0.08).

Regarding alpha power, a similar significant two-way interaction with induction condition was observed. Interestingly, lower alpha power was associated with higher SMS scores during only OM (*b* = −1.74, *SD* = 0.51,* t* = −3.41, *p* < 0.001) but not FA (*b* = −0.01, *SD* = 0.49,* t* = −0.02, *p* = 0.98) compared to C. This interaction was likewise qualified by a three-way interaction involving region, such that the association between alpha power and SMS in the posterior region was significantly more positive for both FA (*b* = 1.39, *SD* = 0.59,* t* = 2.34, *p* = 0.02) and OM (*b* = 1.84, *SD* = 0.62,* t* = 2.98, *p* < 0.01) compared to C. Moreover, there was also a trending interaction showing that the negative relationship between alpha power and SMS was strongest in the frontal region during OM relative to C (*b* = −1.14, *SD* = 0.64,* t* = −1.78, *p* = 0.08).

**Fig. 1:**
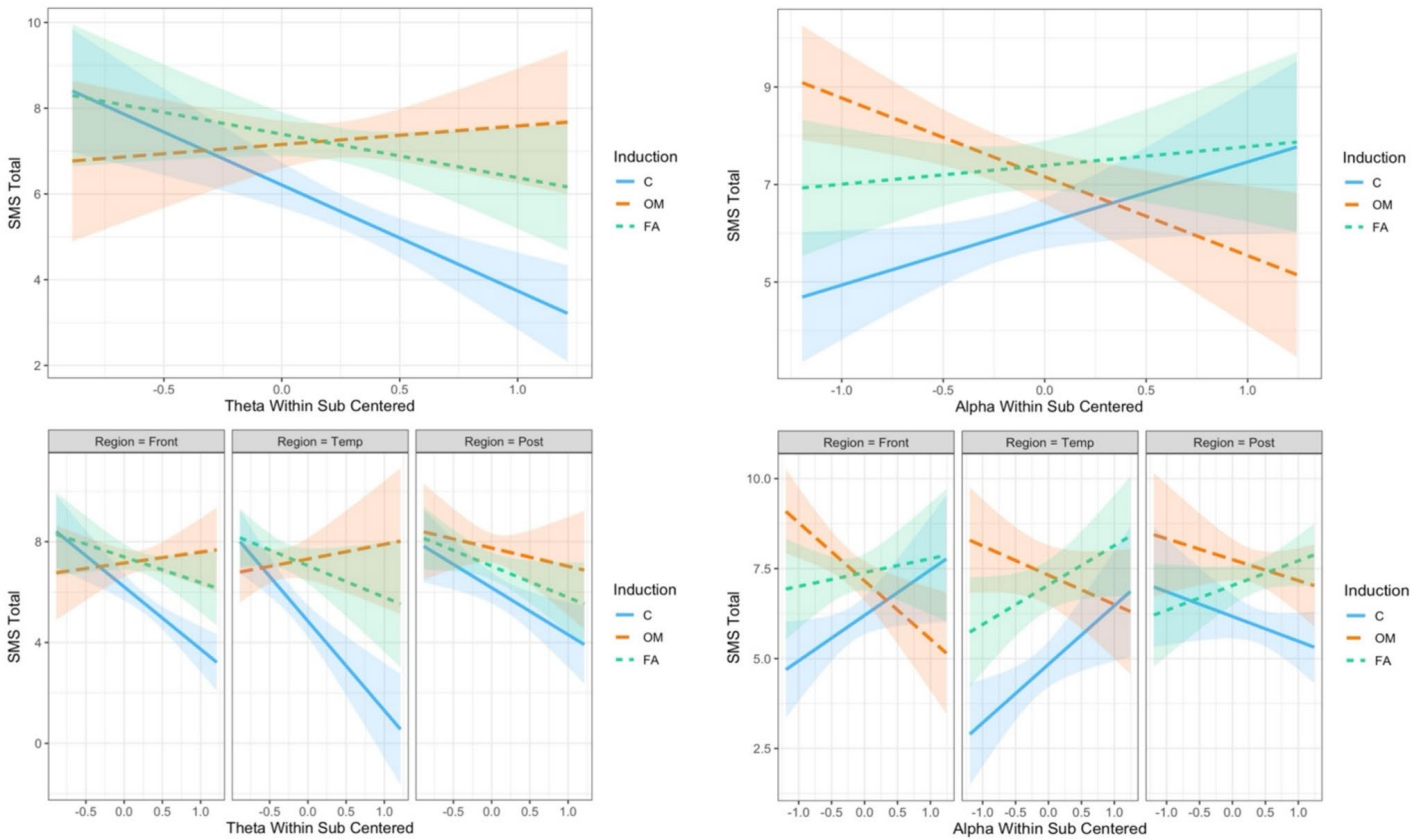
The two-way (above) and three-way (below) interaction plots for theta (left) and alpha (right) spectral power predicting State Mindfulness Scale (SMS) ratings

Finally, to determine whether and the extent to which OM and FA differ from each other, we re-ran the model using FA as the referent condition. The analysis confirmed that the practices were functionally dissociable. Specifically, lower alpha power during OM was selectively associated with higher SMS compared to FA (*b* = −1.73, *SD* = 0.50,* t* = −3.44, *p* < 0.001). Furthermore, there was a trending interaction suggesting that greater theta power during OM was more strongly associated with higher SMS relative to FA (*b* = 1.27, *SD* = 0.71,* t* = 1.78, *p* = 0.08). Visualizations of the key interactions are presented in Fig. 1.

## Discussion

The current study was designed to provide an exploratory “proof-of-concept” demonstration, connecting neural oscillatory activity occurring during focused attention (FA) and open monitoring (OM) meditation practice with actual subjective appraisals of state mindfulness quality. By leveraging a novel within-subject design among novice participants, we directly tested whether alpha and theta power were differentially associated with self-reported state mindfulness across FA and OM, rigorously contrasted against an active control condition. Our findings provide compelling evidence that these two core practices can be characterized via diverging alpha but converging theta patterns of mind-brain relationships. Specifically, we found that reduced alpha power was associated with higher state mindfulness, but uniquely for OM, whereas increased theta power was associated with higher state mindfulness for both practices, but with the effect trending stronger for OM. These relationships were further modulated by scalp topography, highlighting the important role of regional specificity in understanding the neural correlates of distinct mindfulness states.

Contrasting our primary findings with the standard benchmark analyses further highlights the unique value of our approach. The conventional analyses revealed that OM was characterized by less alpha compared to FA, and that both OM and FA were associated with decreased theta power relative to C. Although these results help characterize the effects of the mindfulness inductions on spectral power, they offer an incomplete view of how brain activity is fundamentally related to subjective appraisal of mindfulness. For example, the benchmark analysis showed no main effect of the OM induction on alpha power relative to C, yet our primary analysis revealed a robust negative relationship between alpha power and state mindfulness scores specifically during OM practice. Conversely, the benchmark analysis pointed to reduced theta power in FA and OM relative to C, but our primary analysis suggests that it is *increasing* theta in both conditions which is linked to higher state mindfulness.

Both FA and OM represent attentional mindfulness practices but differ in attentional breadth. The convergence in theta power for FA and OM is consistent with the theory that these mindfulness practices may have overlapping mechanisms for sustained awareness, as they both produced distinct theta activity relative to the control audio (TED talk). However, the divergence in alpha power illustrates how analyses focused solely on condition-level averages may obfuscate important state-dependent brain-mind relationships including differences in narrow and broad attentional focus. The functional significance of alpha power during OM is only discernible by modeling the session-level relationship between neural activity and subjective state mindfulness ratings, thereby reinforcing the utility of shifting analytic focus away from *whether* brain activity differs across different induction conditions, towards *how* activity covaries with the quality of subjective experience within each practice.

Taken together, our findings provide compelling direct empirical evidence that these core meditation practices are characterized by diverging neurophenomenological profiles, which not only differ from a rigorous active control condition, but also from each other. The positive relationship between theta and SMS for both mindfulness conditions confirms the traditional interpretation of theta reflecting engagement in higher cognitive processes, including attention and cognitive control (Cavanagh & Frank, 2014; Magosso et al., 2021). Yet, the selective negative association between alpha power and SMS during OM challenges the common interpretation of enhanced alpha power as a general marker of mindfulness. For novices engaging in OM, lower alpha power may reflect reduced top-down sensory gating and greater receptivity to momentary experience (Klimesch, 1999, 2012).

Although our interpretations remain speculative, they represent a possible alternative that helps contextualize previous mixed results, suggesting that increased theta during FA and OM may reflect overlapping underlying sustained attentional mechanisms. On the other hand, the observed decrease in alpha power during OM may signify a distinct state of mindfulness, perhaps one characterized by more attentional diffusion and openness as opposed to focused concentration. The topographical analyses add further nuance, revealing that the negative alpha-SMS relationship during OM was most pronounced in the frontal region but significantly attenuated posteriorly, whereas the positive alpha-SMS relationship for FA was only significant in the posterior region. Critically, this pattern challenges simplified “one-to-one” interpretations and points toward a dynamic interplay wherein the subjective index of alpha power is not only practice dependent but also region specific.

Beyond this specific pattern of findings, our work raises several important methodological implications. By modeling within-subject variability in spectral power as a predictor of state mindfulness ratings, the approach moves beyond traditional condition or group-level comparisons. Indeed, our results provided a compelling demonstration of this key point: namely, that the nuanced brain-mind relationships revealed in our primary analyses were absent in the conventional benchmark analyses of mean power differences. Rather than assuming a direct correspondence between spectral power and mindfulness, our analysis illustrates how session-level fluctuations can be used to explicitly test both the presence and nature of this relationship. This strategy permits a more precise means of grounding established neural signals within a well-controlled experimentally manipulatable phenomenological context, offering a potentially valuable building block towards refining the collective methodology of contemplative neuroscience.

Indeed, our findings demonstrated the promise of this methodological approach for advancing the basic science of mindfulness. The finding that alpha and theta power exhibit differentiable relationships to subjective mindfulness quality across distinct guided meditation practices established empirical support for the intuitive, yet under-tested, perspective that the functional significance of neural oscillations is context-dependent. The selective negative association between alpha power and state mindfulness during OM, contrasted with the positive association observed for theta power during both FA and OM, suggests that activity within these frequency bands is not simply redundant markers of a unitary “mindful state”. Instead, they appear to reflect diverging attentional processes, and the degree to which they relate to subjective experience seems contingent upon the particular features and demands of the meditation practice. Taken together, our findings support the growing movement to conceptualize mindfulness not as a unitary construct, but as an operationalizable family of discrete states and practices (Davidson & Kaszniak, 2015; Lin et al., 2022; Lutz et al., 2008, 2015; Van Dam et al., 2018), underscoring the importance of explicitly accounting for the specific type of meditation when interpreting neural activity.

### Limitations and Future Directions

The proof-of-concept nature of this study necessitates consideration of its limitations, which in turn point to potentially important future directions. First, the findings are based on a relatively modest sample size (87 sessions of data across 29 participants), and while useful for providing a proof-of-concept demonstration, will need replication to establish their generalizability. Second, our reliance on the State Mindfulness Scale, administered retrospectively at the end of each session, is susceptible to recall bias and fundamentally reduces assessment to a singular value. This further constrains the temporal granularity of identified mind-brain relationships, necessitating averaging spectral power indices across the full 10-min induction to maintain temporal alignment with SMS scores. Future work utilizing repeated experience sampling probes within a session could better characterize the temporal dynamics of practice. Furthermore, the SMS is not designed to provide highly granular differentiation between the diverse experiential and metacognitive dimensions of meditation. Consequently, future studies may benefit from incorporating more phenomenologically oriented approaches, such as the Lyon Assessment of Meditation Phenomenology (LAMP; Abdoun et al., 2025), or Temporal Experience Tracing (Jachs et al., 2022; Lewis-Healey et al., 2024), which may offer a richer and more detailed characterization of meditation experience across multiple domains (e.g., mental content, motivation, meta-awareness).

Although an important strength of our methodology was to use hierarchical modeling to test for how the SMS-power relationship varies by region across induction, our primary analytic approach modeled regional power as nested within sessions, resulting in multiple observations per session. While this structure is standard for testing regional moderation, the repeating observations may underestimate standard errors, potentially inflating statistical significance. Thus, we conducted supplemental sensitivity analyses using an alternative model specification with two-way region x induction interactions that treat regional power values as separate predictors, effectively unnesting region and reducing the number of repeated observations within session (see OSF for full model output). Importantly, the sensitivity analyses confirmed the robustness of our core finding that frontal and posterior regions exhibit opposing patterns of spectral power-SMS relationships during the meditation practices, demonstrating that the region-specific patterns reported here are unlikely a sole product of the nested model structure.

Additionally, while our analyses allowed us to contrast FA and OM states with an active control condition, the processes elicited during the active control require their own consideration and scrutiny. In this case, the control condition was a narrative podcast episode (TED talk) that differed considerably in content, structure, and engagement relative to the two guided inductions. These qualitative differences were clearly reflected in the manipulation check findings, in which significantly stronger engagement, interest, emotional reactions, arousal, understanding and learning occurred, relative to FA and OM. These differing engagement profiles may have contributed to some of the observed differences in spectral power among conditions. Other work examining meditation-related spectral power effects has utilized “instructed mind-wandering” as an active control condition (Braboszcz et al., 2017; Cahn et al., 2010). Thus, it would be worth investigating whether the observed results would replicate if such a condition were implemented in the current design as a parallel guided active control induction.

Furthermore, we instructed participants to keep their eyes open during the audio inductions to mitigate sleepiness and vigilance-related confounds, which we acknowledge diverges from the more prevalent eyes-closed induction paradigms. Consequently, the present findings are not directly comparable to the eyes-closed literature and may introduce limitations to the scope of interpretability and generalizability. In particular, the eyes-open condition may interact with the distinct attentional demands of the two practices. OM encourages broad receptivity to all arising stimuli, which inherently increases visual sensory input from open eyes, a process known to suppress posterior alpha power. In contrast, FA involves internalization of awareness on the breath, which may restrict visual input processing and thereby attenuate suppression of alpha power.

It is thus possible that the observed divergence in alpha power could partially reflect differences in visual engagement rather than meditation state quality alone. However, it is unlikely that the results reported here are solely an artifact of visual processing demands, as significant functional carry-over effects on subsequent cognitive and emotional tasks were also observed (Lin et al., 2024a, b). These neurobehavioral findings suggest that the induction protocol produced a genuine state shift that persisted beyond the immediate period of visual exposure during the induction. To fully disentangle these factors, future research should directly compare eyes-open and eyes-closed protocols to determine the extent to which such procedural differences influence the patterns reported here.

More generally, our findings suggest that an important future direction for research will be to conduct additional studies investigating eyes-open meditation practices, so that these can be more effectively compared with those involving eyes-closed practice. Indeed, in a recent review paper on FA meditation, only 6 out of the 49 studies reviewed included an eyes-open practice condition (Lieberman et al., 2025). It is well-established that under eyes-closed conditions alpha power can dramatically increase, and it is thus challenging to disentangle whether these effects relate to a change in mental state associated with the meditation practice itself, or to the increased likeliness of drowsiness that frequently accompanies sustained eye-closed periods. This confounding effect may be particularly acute in studies of novice meditators, who have reduced experience with sustaining an alert meditative state while maintaining a closed-eye practice. In the current study, we avoided these confounds by requiring participants keep their eyes open across all conditions, thus minimizing drowsiness-related confounds, while also keeping the conditions tightly equated, so that condition-related differences could be identified. Nevertheless, because of the scarcity of eyes-open findings in the literature, it is challenging to make statements regarding the generalizability of our findings due to the predominance of eyes-closed practices. Thus, we encourage investigators to consider including eyes-open practices or conditions in their study designs, particularly in EEG studies conducted with novices, in order to effectively re-weight the imbalance that is currently present in this literature.

The state induction protocol, while affording rigorous within-subject comparisons, is limited in providing only a static representation of mind-brain relationships. Consequently, the present findings delineate the potential neural *correlates* of a meditation state in novices, but not the neural *mechanisms* of skill acquisition that would unfold with repeated practice. For example, although our results selectively associate lower alpha power with higher state mindfulness during OM, this does not imply that reductions in alpha power are therefore a mechanism through which mindfulness quality improves across OM training. Similarly, our results do not imply the convergence of theta activity during FA and OM is guaranteed to remain that way if participants engage in mindfulness training of these practices. The distinction between such a state correlate and a training dynamic is fundamentally important, and each requires distinct research designs and analytic approaches to properly investigate. With that said, the current study provides a valuable empirical and methodological foundation upon which larger-scale longitudinal research can be built.

Toward this end, our team is already working to address these limitations. We recently completed a time-intensive, longitudinal mindfulness study (White et al., 2024), that tracked novice participants through 24 EEG-recorded practice sessions across 8 weeks of both FA and OM training. This intensive repeated-sampling design is specifically tailored to investigate, and distinguish, the neural and subjective training trajectories of FA and OM. By collecting both EEG and self-report meditation data at each session, we are well-equipped to apply more advanced longitudinal statistical techniques to model how session-to-session changes in regional spectral power activity prospectively relate to changes in state mindfulness quality across the two practices (Lin et al., 2026). The present work therefore serves as an essential conceptual and methodological anchor for this next phase of research, providing a clear state-based framework that grounds the analysis and interpretation of changes in neural activity to their subjective and phenomenological context.

## Supplementary Information

Below is the link to the electronic supplementary material.

ESM 1 (DOCX 23.0 KB)

## Data Availability

All data, materials, protocols, and analysis code, including full model specifications and output, are available in the OSF repository (https://osf.io/uv9yn/).

## Declarations

### Use of Artificial Intelligence

Artificial intelligence was only used for adjusting R code for figure formatting.
