# Supplementary material for: Meditation-Specific Neural Predictors of State Mindfulness During Eyes Open Meditation
Source: Mindfulness (N Y). 2026 May 11;17(7):1969–81. doi: 10.1007/s12671-026-02850-6 (PMC13385244; doi:10.1007/s12671-026-02850-6)
Supplement: Supplementary file 1 — (DOCX 23.0 KB) [file 12671_2026_2850_MOESM1_ESM.docx]

Supplemental Sensitivity Analyses

*Spectral power influences on state mindfulness by region*

To address reviewer concerns about nested observations, we conducted supplemental analyses using an alternative model. Rather than nesting region within sessions, this approach unnests region and treats regional spectral power as separate predictors. This “flattened” structure provides more conservative standard errors estimates but does not permit testing of three-way interactions.

The model is specified as: $SMS \sim1 + Induction Condition*Frontal Alpha + Induction Condition*Frontal Theta+ Induction Condition*Temporal Alpha + Induction Condition*Temporal Theta+ Induction Condition*Posterior Alpha + Induction Condition*Posterior Theta+ (1 | Subject).$

Mirroring the primary analyses, results show the association between alpha power and SMS in the posterior region was significantly more positive for both FA (*b* = 2.77, *SD =* 0.79, *t* = 3.51, *p <* 0.001) and OM (*b* = 2.68, *SD =* 1.01, *t* = 2.64, *p =* 0.01) compared to C. The relationship between frontal alpha power and SMS was significantly more negative for OM (*b* = -3.90, *SD =* 1.22, *t* = -3.21, *p <* 0.001) and FA (*b* = -2.84, *SD =* 1.13, *t* = -2.52, *p =* 0.01) relative to C.

For theta power, results show trending two-way interaction involving the frontal and posterior regions for both FA and OM relative to C. Specifically, for both meditation conditions, increases in frontal theta power was associated with increases in subjective mindfulness (OM: *b* = 2.84, *SD =* 1.45, *t* = 1.95, *p =* 0.05; FA: *b* = 2.59, *SD =* 1.48, *t* = 1.74, *p =* 0.08) whereas decreases in posterior theta for both OM (*b* = -3.67, *SD =* 1.94, *t* = -1.89,  *p =* 0.06) and FA (*b* = -2.94, *SD =* 1.54, *t* = -1.90, *p =* 0.06) were related to increases in SMS.

Using FA as a referent, this model showed no significant differences between FA and OM for either alpha or theta power.

These supplemental analyses are consistent with the primary model’s core finding: Spectral power-SMS relationships show opposing patterns for OM and FA, in both spectral power bands comparing the frontal and posterior regions relative to C (i.e., for the meditation conditions, increased SMS was associated with both decreases in frontal alpha power and posterior theta power, as well as increases in both posterior alpha and frontal theta power).

This flattened model, however, is not without its own limitations. While the power x region relationships are strengthened in this model, the pure induction-level effects are weakened, likely due to the addition of separate six region x induction interaction terms. Nevertheless, given that the region-specific patterns converge across both models, the results from the primary nested model are not solely attributable to the repeating data structure of the original analyses.

| **Table 1.** *Model output for SMS moderated by alpha and theta power* | | | | |
| --- | --- | --- | --- | --- |
| **Model** | **Fixed Effects** | **Estimate (*SD*)** | ***t*-value** | ***p*-value** |
| C as referent | Intercept | 5.87 (0.93) | 6.28 | <0.001* |
|  | Induction OM | 1.38 (1.22) | 1.13 | 0.26 |
|  | Induction FA | 1.02 (1.07) | 0.95 | 0.34 |
|  | Alpha Region Frontal | 1.73 (0.98) | 1.76 | 0.08 |
|  | Theta Region Frontal | -2.35 (1.02) | -2.30 | 0.02* |
|  | Alpha Region Temporal | 1.05 (0.81) | 1.30 | 0.19 |
|  | Theta Region Temporal | -3.11 (1.68) | -1.85 | 0.07 |
|  | Alpha Region Posterior | -1.91 (0.77) | -2.49 | 0.01* |
|  | Theta Region Posterior | 2.18 (1.46) | 1.49 | 0.14 |
|  | Induction OM:Alpha Region Frontal | -3.90 (1.22) | -3.21 | <0.001* |
|  | Induction FA:Alpha Region Frontal | -2.84 (1.13) | -2.52 | 0.01* |
|  | Induction OM:Theta Region Frontal | 2.84 (1.45) | 1.95 | 0.05 |
|  | Induction FA:Theta Region Frontal | 2.59 (1.49) | 1.74 | 0.08 |
|  | Induction OM:Alpha Region Temporal | -0.82 (1.12) | -0.72 | 0.47 |
|  | Induction FA:Alpha Region Temporal | 0.42 (1.06) | 0.40 | 0.69 |
|  | Induction OM:Theta Region Temporal | 3.45 (2.14) | 1.61 | 0.11 |
|  | Induction FA:Theta Region Temporal | 2.08 (2.07) | 1.01 | 0.32 |
|  | Induction OM:Alpha Region Posterior | 2.68 (1.01) | 2.64 | 0.01* |
|  | Induction FA:Alpha Region Posterior | 2.77 (0.79) | 3.51 | <0.001* |
|  | Induction OM:Theta Region Posterior | -3.68 (1.94) | -1.89 | 0.06 |
|  | Induction FA:Theta Region Posterior | -2.94 (1.55) | -1.90 | 0.06 |
| Note. SMS = State Mindfulness Scale, FA = Focused Attention, OM = Open Monitoring. Alpha and theta values are within-subject centered, calculated by subtracting the alpha and theta values for each induction and region from the respective, subject-level mean. * denotes *p*-values < 0.05. | | | | |
